# Low-Molecular-Weight Organic Acid as an Alternative to Promote the Rooting of Persimmon Rootstock Shoot Cuttings

**DOI:** 10.3390/plants13233440

**Published:** 2024-12-08

**Authors:** Jingjing Geng, Chi Zhang, Shaoning Deng, Bowei Liu, Mengye Cheng, Xiuhong An, Hongxia Wang, Wenjiang Wang

**Affiliations:** 1 College of Horticulture, Hebei Agricultural University, Baoding 071000, China; sygjj@hebau.edu.cn (J.G.); 15031175765@163.com (S.D.); wangwj65@126.com (W.W.); 2 National Engineering Research Center for Agriculture in Northern Mountainous Areas, Baoding 071000, China; zhangchi244@163.com (C.Z.); 15297359806@163.com (B.L.); 15233210509@163.com (M.C.)

**Keywords:** *Diospyros lotus* L., organic acids, adventitious root, anatomical structure, endogenous hormones

## Abstract

Organic acids are naturally present in plants and exert a positive influence on plant development, which justifies surveying their potential effect on adventitious root (AR) formation. In this study, 0.0298 mol/L (4000 mg/L) of malic acid and 0.0267 mol/L (4000 mg/L) of tartaric acid were used to explore the effects of low-molecular-weight organic acid on the rooting of persimmon rootstock *Diospyros lotus* L. during cutting propagation. After organic acid treatment, the rooting percentage and the survival rate significantly increased, accompanied by a greater development of lateral roots. Anatomical analysis revealed that *Diospyros lotus* L. exhibits characteristics that induce root primordia, and organic acid treatment can enhance the differentiation of root primordia. Furthermore, treatment with organic acid led to a substantial decrease in soluble sugar and starch contents, along with a slight increase in soluble protein content during early cutting stages. Additionally, the indole-3-acetic acid (IAA) content peaked in the early stages of AR formation and was significantly higher than that of the control, while abscisic acid (ABA) levels exhibited the opposite trend. Comparatively, gibberellic acid (GA_3_) remained at extremely low levels throughout the rooting process in the organic acid groups compared to the control. In conclusion, the current study uncovers the anatomical structure over time during AR formation, revealing the dynamic changes in the related main nutrients and hormones and providing new ideas and a new practical approach for improving root regeneration in persimmon rootstock cuttings.

## 1. Introduction

With the rapid development of modern fruit cultivation systems, rootstocks have been playing an increasingly important role in production [1]. Rootstocks have an important effect on the physiological characteristics and the growth and development of grafted plants, such as fruit quality and productivity, the mature period, plant height, crown size, disease susceptibility, and tolerance against environment stresses [2,3,4]. The vast majority of the most highly produced perennial fruits and nuts need to be grafted onto suitable rootstocks [5]. Therefore, a superior variety of rootstock would be indispensable for modern agricultural production. Clonal rootstocks are widely used during cultivar breeding and selection processes because of their stable genetic background. Cutting propagation is an important clonal propagation method in fruit trees, in which adventitious root formation is the key physiological step for the survival of isolated plants [6]. Therefore, finding a low-cost, environmentally friendly treatment with a good rooting effect is very important for rootstock cutting propagation.

The ability to evoke adventitious roots (ARs) in stem cuttings is dependent on numerous external and endogenous factors, such as environmental conditions, nutrient metabolism, and hormone homeostasis [7,8,9]. In addition, the differences vary by cultivar and are also considered an important factor [10], making the improvement of AR formation a research hotspot and a potential area for advancement in the fruit tree industry. Plant growth regulators are the most commonly used agents to promote rooting in cutting propagation, especially for difficult and slow-to-root species, which can greatly benefit from their application, with improved rooting and survival ratios [11]. Nishimura et al. [12] reported that the rooting rate of cuttings of the persimmon dwarfing rootstock ‘Hourakudai’ was effectively improved after 5500 ppm 1-napthaleneacetic acid (NAA) treatment. Wu et al. [13] found that the hardwood cuttings of persimmon rootstock Yalin 6 under appropriate concentrations of indole-3-butyric acid (IBA) or indole-3-acetic acid (IAA) had a higher rooting rate and sprout growth compared with control plants. However, the application of auxins often has a high cost, potential toxicity risks, and side effects, such as an increased ethylene content in the upper part of the cuttings [14]. Despite adequate rooting, auxins usually inhibit subsequent bud germination and new shoot development in cuttings [14,15,16]. Thus, it is urgent to explore potential new chemical biology alternatives and advanced technologies.

Low-molecular-weight organic acids are widely present in the roots, leaves, and fruits of plants, serving as primary flavor and nutritional components [17]. They not only participate in physiological metabolic processes, such as nutrient absorption and stomatal opening in plants, but they also act as connectors for various cellular metabolic pathways, thereby exerting a positive influence on plant growth and development [18]. It has been reported that exogenous malic acid and citric acid, at a reasonable dose, could function as a priming agent in promoting seed germination in *Solanum pseudocapsicum* under copper stress [19]. A similar phenomenon was observed in *Leymus chinensis*, where a higher germination percentage was observed at suitable salicylic acid concentrations [20]. Hu et al. [21] demonstrated that external citric acid induced the accumulation of endogenous citric acid in leaves and increased root activity under heat stress. Moreover, in rose ‘Sherbet’, spraying citric and malic acid on the branches and leaves before cutting improved the rooting success ratio [22]. Compared with traditional rooting agents, low-molecular-weight organic acids, as natural compounds, have a lower toxicity to plants, do not readily cause drug damage, and are safer. At the same time, they can also reduce the dependence on chemicals during agricultural production, thereby reducing production costs. The research on and application of organic acids are of great significance to the development of environmentally friendly, efficient, and safe agricultural technology. The effect of organic acids on rooting performance during the cutting process has barely been explored in fruit trees.

Persimmon (*Diospyros kaki* Thunb.) is the representative fruit tree species within the *Diospyros* genus, owing to its substantial economic value. It is believed to have originated in China and is extensively cultivated across Asian countries [23,24]. Root regeneration in cuttings is crucial for large-scale propagation of persimmon rootstocks. However, persimmon is a difficult-to-root cultivar with regard to shoot cutting. In a previous study, we first demonstrated that in addition to being a plant growth regulator, citric acid also significantly enhances the rooting rate of persimmon softwood cuttings [25]. To further explore the influence of low-molecular-weight organic acids on the rooting of persimmon cuttings, the effects of malic and tartaric acid on rooting during cutting propagation were systematically studied using L938 (*Diospyros lotus* L.), a widely compatible persimmon rootstock used in China. The rooting process of softwood cuttings was observed anatomically after different treatments; meanwhile, the changes in nutrients and endogenous hormones were determined to explore the physiological changes of L938 during cutting rooting under organic acid treatment. This study presents an eco-friendly alternative to traditional rooting agents and shows that organic acids enhance rooting rates and lateral root formation, offering a sustainable solution for persimmon propagation. 

## 2. Results

### 2.1. Low-Molecular-Weight Organic Acid Treatment Promotes the Rooting of Persimmon Rootstock L938

**Figure 1:**
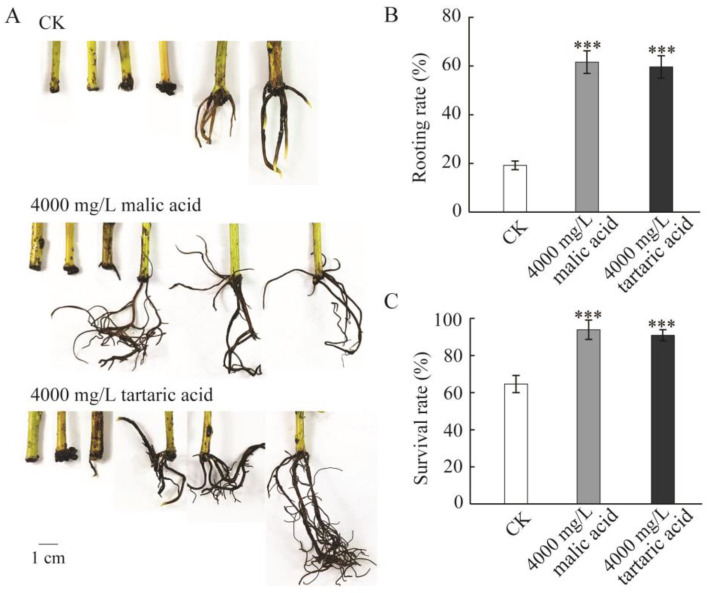
Low-molecular-weight organic acid treatment promotes the rooting of persimmon rootstock L938. (**A**). The morphological changes in adventitious roots of L938 cuttings under different treatments. (**B**). Rooting rate (% of cuttings that developed at least one root). (**C**). Survival rate (% of live cuttings). There were 33 plants per treatment and three replications. Values are the means of three replicates and the error bars represent the standard error. Asterisks indicate a significant difference between the CK and the organic acid treatment in the rooting rate and the survival rate (*** means *p* < 0.001).

To investigate the role of low-molecular-weight organic acids in the rooting of persimmon rootstock cuttings, 0.0298 mol/L (4000 mg/L) of malic acid and 0.0267 mol/L (4000 mg/L) of tartaric acid were used in the experiment. Visible morphological differences in rooting were observed between the organic acid treatment groups and the CK plants (Figure 1A). The rooting percentage and the survival rate in cuttings with malic acid and tartaric acid treatments were significantly higher than those in CK plants (Figure 1B,C). Meanwhile, more lateral roots appeared after organic acid treatment (Figure 1A). These results indicate that organic acid is effective at promoting root growth in persimmon rootstock L938 cuttings.

### 2.2. Anatomical Evaluation of the Rooting Process Under Different Treatments

**Figure 2:**
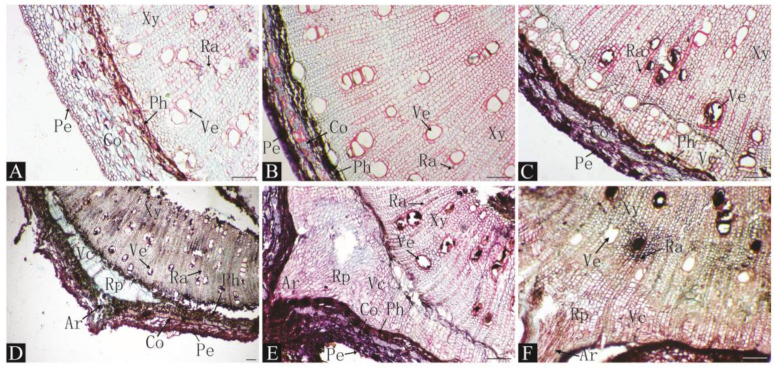
Histological observations of pre-adventitious root initiation stages of L938 cutting under 4000 mg/L malic acid treatment. (**A**). 0 d. (**B**). 10 d. (**C**). 20 d. (**D**). 30 d. (**E**). 40 d. (**F**). 50 d. Rp: root primordium; Ar: adventitious root; Pe: epidermis; Co: cortex; Ph: phloem; ve: vessel; Xy: xylem; Vc: vascular cambium; Pi: pith; Ra: pith ray. Scale bar: 100 μm.

The anatomical structures of each treatment were observed every 10 days during the rooting process after cutting (Figure 2). After malic acid treatment, many meristem cells appeared in the vascular cambium on day 20, which were arranged neatly and tightly, indicating that cambium cells were rapidly growing and differentiating at 10–20 days after malic acid treatment. For the next 10 days, cambium cells continued to grow and formed root primordia. Moreover, several root primordia formed adventitious roots, breaking through the epidermis in this period. After 30–40 days, the root primordia continued to form and develop into adventitious roots; meanwhile, the inner stem tissue started to connect with the adventitious root vascular system, and the root system began to absorb and transport nutrients. After 40–50 days, further elongation of large numbers of adventitial roots occurred, and they subsequently broke through the epidermis. Simultaneously, the vascular system of adventitial roots was linked to xylem pulp rays and directly reached the pulp center.

**Figure 3:**
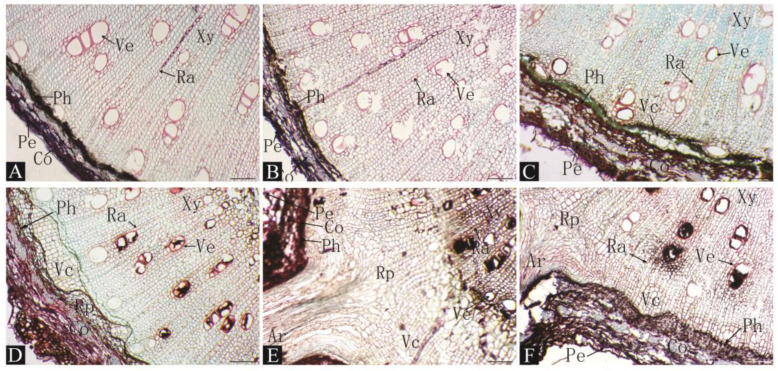
Histological observations of pre-adventitious root initiation stages of L938 cuttings under 4000 mg/L tartaric acid treatments. (**A**). 0 d. (**B**). 10 d. (**C**). 20 d. (**D**). 30 d. (**E**). 40 d. (**F**). 50 d. Rp: root primordium; Ar: adventitious root; Pe: epidermis; Co: cortex; Ph: phloem; Ve: vessel; Xy: xylem; Vc: vascular cambium; Pi: pith; Ra: pith ray. Scale bar: 100 μm.

As shown in Figure 3, the process of adventitious root formation under tartaric acid treatment was similar to that under malic acid treatment; namely, 10–20 days after cutting, cambium cells appeared and gradually grew. After 20–30 days, large numbers of cambium cells differentiated and were arranged closely, and the root primordia began to form. Subsequently, the root primordia formed rapidly and developed into adventitious roots, breaking through the epidermis after 30–40 days. After 40–50 days, the root primordium activity was strong inside of the cuttings, and many adventitious roots formed; meanwhile, the adventitial root vascular bundle connected with the inner tissue of the stem and began to play a physiological role.

**Figure 4:**
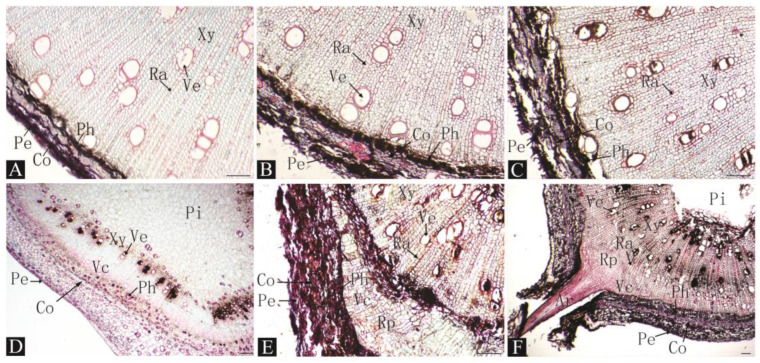
Histological observations of pre-adventitious root initiation stages of L938 cutting under water condition (CK). (**A**). 0 d. (**B**). 10 d. (**C**). 20 d. (**D**). 30 d. (**E**). 40 d. (**F**). 50 d. Rp: root primordium; Ar: adventitious root; Pe: epidermis; Co: cortex; Ph: phloem; Ve: vessel; Xy: xylem; Vc: vascular cambium; Pi: pith; Ra: pith ray. Scale bar: 100 μm.

For the CK treatment, root primordia did not form until 40 days after cutting, indicating the formation of cambial cells, and root primordia growth was significantly slower than after organic acid treatment. It took 50 days for the adventitial root to break through the epidermis (Figure 4).

By observing the anatomical structure of a stem cross-section after cutting and rooting, we found that before cutting, the structure from the outside to the inside was as follows: periderm, cortex, phloem, xylem, and pith. There were medullary rays and ducts in the xylem, and no latent root primordia were found. Therefore, we concluded that the type of cutting rooting in date plums induced rooting. Comparing the time at which cambium and root primordia occurred for each treatment, it was found that a small number of cambium cells appeared in the control group after 20 d, and root primordia formed until 40 d after cutting. However, many cambium cells appeared 10–20 d after cutting with organic acid treatment, and root primordia formed within 30 d after cutting. These results show that organic acid treatment can promote the formation of cambium cells and root primordia in cuttings.

### 2.3. Dynamic Changes in the Contents of Soluble Sugar, Starch, and Soluble Protein During the Rooting Process of Cuttings

**Figure 5:**
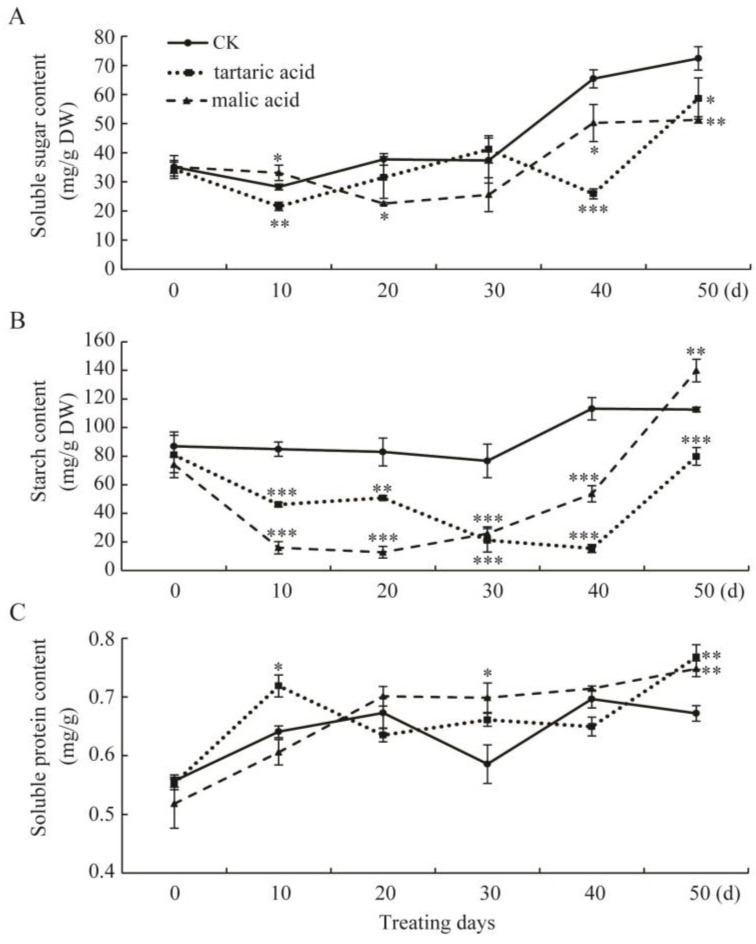
Dynamic changes in the contents of soluble sugar (**A**), starch (**B**), and soluble protein (**C**) during the rooting process of cuttings. Values are means of three replicates, and the error bars represent the standard error. Asterisks indicate significant difference between CK and organic acid treatments (* means *p* < 0.05, ** means *p* < 0.01, *** means *p* < 0.001).

To study the effects of organic acid treatment on nutrient changes during rooting, the contents of soluble sugar, starch, and soluble protein were determined, as shown in Figure 5A. In the tartaric and malic acid treatments, the soluble sugar content decreased to its lowest at 10 and 20 days, respectively, and the minimum values were significantly lower than those in CK plants. Meanwhile, after 40–50 days, the soluble sugar content was significantly higher after CK treatment than after organic acid treatment, which might be due to the lower amount of adventitious roots and the lower energy consumption in the control treatment group, in addition to the large amount of nutrients produced via shoot photosynthesis during this period.

Compared with CK plants, the starch content in treated cuttings was significantly affected by organic acid (Figure 5B). In CK plants, it changed slightly during the cutting process, while the starch content of cuttings treated with organic acid fluctuated greatly, decreasing first and then increasing. The starch content decreased to a minimum value after 20 and 40 days under tartaric acid and malic acid treatment, respectively, and it was significantly lower than the values in CK plants. Afterwards, this value increased rapidly.

The soluble protein content remained on a similar level for all treatments (Figure 5C). During the first 20 days, a small increase was observed in all treatments compared to the initial value. For CK, a decreasing trend was observed on day 30, and then no significant change was observed until day 50, after which the soluble protein contents in the two organic acid treatment groups were higher than those in the CK plants.

### 2.4. Dynamic Changes in the Contents of Endogenous Phytohormones Zeatin (ZT), Abscisic Acid (ABA), Indole-3-Acetic Acid (IAA), and Gibberellic Acid (GA_3_) During the Rooting Process of Cuttings

**Figure 6:**
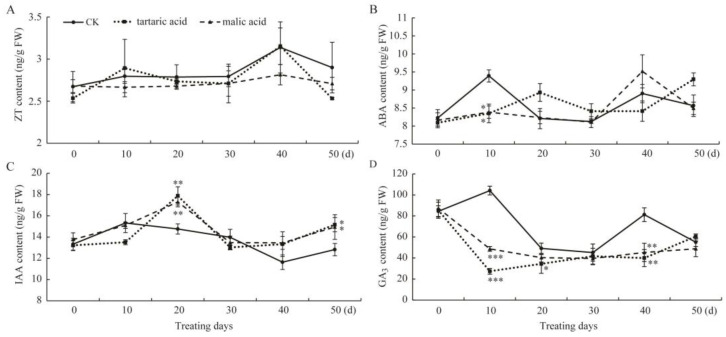
Dynamic changes in the contents of four endogenous hormones during the rooting process of cuttings. (**A**). ZT content. (**B**). ABA content. (**C**). IAA content. (**D**). GA_3_ content. Values are means of three replicates, and the error bars represent standard errors. Asterisks indicate significant differences between CK and organic acid treatments (* means *p* < 0.05, ** means *p* < 0.01, *** means *p* < 0.001).

Hormones play an important role in the development of adventitious roots in plants [6]; thus, we determined the endogenous hormone contents in cuttings during rooting. The results show that the ZT content was not visibly different between organic acid and CK treatment groups (Figure 6A). It remained essentially the same as the initial level for all treatments during the whole cutting process, indicating that organic acid treatment has no influence on ZT content in rooted L938 cuttings.

ABA can inhibit cell growth and elongation, and it mainly functions as a rooting inhibitor during the rooting process [26]. As shown in Figure 6B, the ABA content in CK plants was notably higher on day 10 than that after organic acid treatment, indicating that these two organic acids could inhibit ABA formation in the early stages of cutting, which is beneficial for cutting rooting. Over the next 40 days, the ABA content was maintained at a relatively stable level, and there were no significant differences among all treatments. These results indicate that organic acid treatment inhibits ABA formation in the early stages of cutting, which is beneficial for cutting rooting.

IAA can accelerate cell division to promote the formation of adventitious roots and speed up cell growth in the root elongation zone to stimulate root elongation [27]. The IAA content increased in all treatments during the first 10 days. After that, the IAA levels in CK plants began to decline, with the minimum value observed on day 40, and then they showed a small rebound on day 50. However, in the two organic acid treatment groups, IAA levels kept increasing until reaching a peak on day 20, and they were significantly higher than those in CK plants. They then decreased until day 40 and began to rise again on day 50 to a level significantly higher than that in CK plants (Figure 6C).

The content of GA_3_ was also remarkably affected by tartaric acid and malic acid treatments (Figure 6D). Completely contrasting trends were observed during the whole rooting process between CK plants and plants that were treated with the two organic acids. In the early stages of cutting, the GA_3_ content in CK plants continued to increase until it reached the maximum value on day 10, while in the two organic acid treatment groups, it kept falling and was significantly lower than that in the control at this time. Over the next 10 days, the GA_3_ level in CK plants started to decline, and on day 20, it was slightly higher than that of the organic acid groups. In the middle and late stages of cutting, the GA_3_ contents in two organic acid treatment groups remained relatively stable, while an increase occurred in the CK plants after 30–40 days, followed by a decrease; on day 50, no differences were observed among all treatments.

## 3. Discussion

Cutting is crucial for the vegetative propagation of fruit trees, and plant growth regulators (e.g., IBA and NAA) are often used to promote the adventitious rooting of asexually propagated cuttings. However, due to the high cost and potential toxicity risks associated with the excessive use of plant growth regulators for both animals and humans, there is an urgent need to explore natural products and organic rooting substances as alternatives for plant growth regulators in agricultural production [28]. Low-molecular-weight organic acids not only serve as a carbon source for plant growth but also regulate auxin synthesis, alter the rhizosphere environment and microbial activity, control fleshy fruit acidity, and impact plant adaptability to environmental stressors [17,29,30]. Low-molecular-weight organic acid is considered to be an exogenous additive with great agronomic potential for improving the stress tolerance of plants, but it is rarely used as an agent in cutting propagation. In an earlier study, we established that citric acid could significantly enhance the rooting rate of persimmon softwood cuttings [25]. Additionally, compared to the suppression of bud sprouting by plant growth regulators, organic acid treatment is more beneficial for the initiation and elongation of new shoots in cuttings (data not presented). Here, we further proved that malic and tartaric acid also play a key role in promoting cutting rooting, leading to more lateral roots compared with the control. These results suggest that low-molecular-weight organic acids are effective agents for the successful cutting of persimmon rootstocks.

In this study, no latent root primordia were found in the anatomical observations of the treated cuttings (Figure 2, Figure 3 and Figure 4), indicating that the rooting type of the persimmon rootstock date plum L938 is induced rooting. The origin of root primordia in date plum cuttings is cells with a strong division ability that are located between the xylem and the phloem in the vascular cambium layer. Additionally, little callus tissue was formed at the base of the cuttings, and rooting occurred in the epidermal region, which is a similar observation to that made by Izhaki et al. for *Diospyros virginiana* rootstocks [16]. Persimmon is a difficult and slow-to-root species via cutting similar to cashew and chestnut [31]. Anatomical observations also identified a continuous ring of thick-walled tissue within the cortex near the phloem layer, which is suspected to be one of the reasons for the complex and slow rooting of persimmon via cutting. A similar phenomenon was noted in the anatomical observations of Feijoa [32]. Treating cuttings with organic acid resulted in noticeable changes in cambial cell differentiation within 10–20 days post planting compared to the gradual changes observed under control conditions over 30–40 days, indicating that organic acid treatment can promote the differentiation of adventitious root-forming cells. Furthermore, it was noted that after 50 days, surviving cuttings without visible roots exhibited both root primordia and adventitious roots in stem cross-sections, indicating a constant formation of adventitious roots throughout the cutting process. These cuttings have the potential to root even during later stages of cutting if sufficient time is provided.

Nutrient levels in cuttings influence the rooting process [33]. Organic acid treatment can regulate the physiological metabolism of cuttings and promote nutrients to facilitate adventitious root formation. The content of soluble sugars and starch in cuttings generally exhibited a “decrease–increase” trend during the entire cutting period (Figure 5A,B). This may be attributed to the fact that during the early stages of cutting, when cuttings are separated from the mother plants, the formation of root primordia, leaf primordia, and adventitious roots requires a substantial amount of nutrients and a significant amount of soluble sugars and starch is consumed to provide energy for cutting growth, which reduces their contents. In later stages, as adventitious roots continue to grow and new shoots and leaves begin to photosynthesize, nutrient accumulation exceeds nutrient consumption, leading to a gradual increase and stabilization in soluble sugar and starch contents within the cuttings. The trends in soluble sugar and starch contents after treatment with different organic acids vary in intensity and duration over time (due to the stimulation effect), which is associated with the different effects of the treatments on cuttings. The results of this study indicate that treatments causing greater reductions in soluble sugar and starch contents during early cutting stages with longer durations are more conducive for promoting adventitious root formation, resulting in higher rooting rates. Meanwhile, this study revealed an overall slight upward trend in soluble protein contents within treated persimmon rootstock cuttings during the rooting process (Figure 5C). A similar phenomenon in the early stages was observed by Chen et al. [34] in a study on pine cutting. Additionally, our study found that fluctuations in soluble protein content were smaller compared to fluctuations in carbohydrates, with significantly lower amounts of proteins than carbohydrates, which is consistent with the results of Wang et al. [35].

The formation of adventitious roots in plants is elegantly controlled by endogenous hormone metabolism, which is a complex multi-hormonal interaction process. The production of endogenous hormones in cuttings is initiated by axillary buds, and these hormones are subsequently transported to the base, where they regulate the formation of adventitious roots [36]. Among these hormones, auxin plays a key role in regulating the process of adventitious root formation in cuttings [37]. During cutting rooting of most plant species, IAA contents show a significant positive correlation with stronger root formation abilities, such as in *Morus alba* L. [27], *E. grandis* [38], and *Platycladus orientalis* [7]. However, no prominent effect on adventitious root development was observed in *E. globulus* [38]. Our data highlight that the IAA content in the persimmon rootstock date plum cuttings exhibits an “up–down–up” trend, while the IAA content in the control group fluctuates with a relatively smaller amplitude (Figure 6C). Organic acids can increase IAA levels and promote root primordium formation during the induction period of adventitious roots, and the IAA level decreases during the elongation period. However, it rises again in the late stages of cutting, further demonstrating that the cuttings that survive until the later stages are still forming adventitious roots after being treated with organic acids, which is consistent with the results in *Eucommia ulmoides* [39].

ZT is a kind of cytokinin, and there is significant controversy regarding its impact on the rooting of cuttings. According to Ma et al. [40], low concentrations of ZT inhibit cutting rooting, while Liu et al. [7] believe that high levels of ZT are conducive to the formation of calluses and the differentiation of root primordia in the early stages. They also concluded that a reduction in ZT levels was beneficial to the elongation of adventitious roots. In the current study, we found that there was no large difference between the ZT content after organic acid treatment and that of the control group during the whole cutting process (Figure 6A), suggesting that ZT has a minimal impact on adventitious root formation in date plum.

ABA has an inhibitory effect on cell growth and is a suppressive plant hormone [26]. Its synthesis and expression of effector genes participate in the inhibition of callus formation, which results in the negative regulation of adventitious root formation [41]. Various studies have reported that low levels of ABA are conducive to root primordium differentiation and root formation [7,27]. In this study, the ABA levels in organic acid groups were significantly lower than those in control plants in the early stages of cutting (Figure 6B), indicating that reduced ABA content in cuttings was conducive to the formation of root primordia, thereby promoting rooting.

GA_3_ can promote cell division and plant stem elongation, while its impact on cutting rooting varies depending on the specific variety and species. A positive relationship between the GA_3_ content and stronger root formation of *M. wufengensis* was confirmed by Wang et al. [42]. El-Banna et al. [36] observed a rising trend in GA_3_ concentration during the early stages of smock tree cutting after treatment with IBA. However, according to Koyama et al. [43], high concentrations of GA_3_ inhibit adventitious rooting and vice versa. As discovered by Chen et al. [27], the GA_3_ contents of two *Morus alba* L. cuttings showed a continuous decreasing trend throughout the rooting process, which is consistent with the results of Mu et al. [44]. In the current investigation, the GA_3_ content of the cuttings in the organic acid treatment group rapidly decreased and was significantly lower than that of the control in the early growth period of 0–20 days, remaining at a lower level throughout subsequent stages (Figure 6D). This indicates that organic acid treatment can effectively reduce GA_3_ levels in cuttings, which is beneficial for promoting root development in date plum softwood cuttings.

## 4. Materials and Methods

### 4.1. Plant Materials

The plant materials (date plum L938) were grown at the experimental base on the East Campus of Hebei Agricultural University (Baoding, China). On 21 June 2022, current-year shoots of 30 cm in length were used as the cuttings for the rooting experiments. The apical young part of the shoots was removed; then, every 2 segments were used as a cutting, which were bevel cut at the base, with 1/2 to 1/4 of the upper segment leaves retained and the lower leaves removed. Then, the prepared segments were mixed evenly and used as cuttings.

### 4.2. Rooting and Sampling of Cuttings

**Figure 7:**
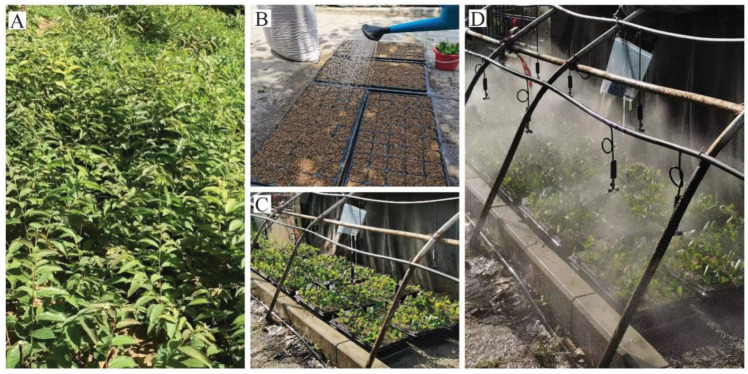
Cutting process of date plum L938. (**A**). Date plum L938 plants used for cutting. (**B**). The substrate was sprayed with 1000 × carbendazim for disinfection. (**C**,**D**). The planted cuttings of date plum L938 placed in the full-light misty seedbed.

The base of the cuttings was soaked in solutions containing 0.0298 mol/L (4000 mg/L) malic acid (99% purity; CAS#6915-15-7, Yuanye^®^, Shanghai, China) and 0.0267 mol/L (4000 mg/L) tartaric acid (99% purity; CAS#133-37-9, Yuanye^®^, Shanghai, China) for 2 h, respectively. Water was used as a control. The treated cuttings were vertically inserted into a hole tray (5 cm in diameter) (Shengli Chemical Technology Co., Ltd., Nanjing, China) that was filled with coarse-grained vermiculite (3~5 mm) as the substrate. About 2/3 of the length of each cutting was submerged in the growth media and then sprayed with 1000 × carbendazim for disinfection. Each treatment included 420 cuttings in a randomized block design with 3 replications. Then, all of the cuttings were placed under an intermittent mist using an automatic spray system. Misting was conducted for 20 s every 12 min in order to keep the cuttings fresh (Figure 7).

At 0 d, 10 d, 20 d, 30 d, 40 d, and 50 d after cutting, 20 samples were randomly collected from each treatment and each repetition. The vermiculite was rinsed off of the samples with clean water before they were labeled and taken back to the laboratory in an ice box. After washing with deionized water and wiping them dry, the bottom (0.5 cm) of the samples was cut off and put into formalin–acetic acid–alcohol (FAA) (a mixture of formaldehyde, glacial acetic acid, and 96% ethanol [16:5:50 (*v*/*v*/*v*)]) for paraffin sectioning. The middle and lower parts of the rest of the cuttings were quickly frozen with liquid nitrogen and ground into a powder before being loaded into a labeled centrifuge tube. Finally, they were placed in a refrigerator at −80℃ until further physiological and biochemical tests. All procedures were performed in a low-temperature environment (in an ice box).

### 4.3. Anatomical Analysis

Anatomical observations of the rooting process were performed using the safranin-fast green method according to Ilczuk and Jacygrad [45] with minor changes. Briefly, the explants (0.5 cm basal segments of the samples) were first fixed in FAA for more than 12 h, followed by dehydration using a graded ethanol–xylene series. Finally, they were embedded in paraffin wax (52–54 °C melting point, Paraplast^®^, Sigma, Tokyo, Japan) [46]. Then, the embedded blocks were serially sectioned (10 μm) using a rotary microtome Leica Model lRM 2235 (Leica Biosystems Nusstoch GmbH, Nussloch, Germany) before being spread out, baked, and then subjected to graded dewaxing and rehydration. After staining with 1% (*w*/*v*) safranin and 0.1% (*w*/*v*) fast green solutions, the sections were sealed with neutral gum and covered with coverslips. Photographs were taken using a SOPTOP CX40 biological microscope (Sunny Instruments, Ningbo, China) and analyzed using MvImage View (3.2.1.20231107) software.

### 4.4. Physiological Measurements

Soluble sugar and starch contents were determined via anthrone sulfuric acid colorimetry [47]. Soluble protein was measured according to Bradford’s method [48]. Endogenous hormone contents were analyzed via high-performance liquid chromatography (PerkinElmer Binary LC Pump, Waltham, Massachusetts, USA) [49]. Absorbance in all measurements was calculated using a spectrophotometer (UV-1800, Shimadzu, Kyoto, Japan), with three replicates for each extract.

### 4.5. Statistical Analysis

All of the experimental data were measured in three biological replicates, and analysis of variance (ANOVA) was performed using the SPSS 18.0 package (SPSS Inc., Chicago, IL, USA). Significant values were discriminated using Tukey’s honestly significant difference (_HSD_) test (*p* = 0.05).

## 5. Conclusions

Low-molecular-weight organic acid can promote the differentiation of root primordia and effectively induce adventitious roots by accelerating the consumption of soluble sugar and starch, increasing IAA, and reducing GA_3_ levels in persimmon rootstock cuttings.

## Data Availability

The remaining data presented in this study are available upon request from the corresponding author. The remaining data are not publicly available due to privacy.
